# Supplementary material for: LXRα Regulates ChREBPα Transactivity in a Target Gene-Specific Manner through an Agonist-Modulated LBD-LID Interaction
Source: Cells. 2020 May 13;9(5):1214. doi: 10.3390/cells9051214 (PMC7290792; doi:10.3390/cells9051214)
Supplement: Supplementary file 1 [file cells-09-01214-s001.pdf]

Supplementary Data

LXRα regulates ChREBPα transactivity in a target gene-specific manner through an agonist-modulated LBD-LID interaction

Qiong Fan[1], Rikke Christine Nørgaard[2], Ivar Grytten[3], Cecilie Maria Ness[2], Christin Lucas[2], Kristin Vekterud[1], Helen Soedling[2], Jason Matthews[2], Roza Berhanu Lemma[4], Odd Stokke Gabrielsen[4], Christian Bindesbøll[1], Stine Marie Ulven[2], Hilde Irene Nebb[2], Line Mariann Grønning-Wang[2] and Thomas Sæther[1].

[1]Department of Molecular Medicine, [2]Department of Nutrition, Institute of Basic Medical Sciences, [3]Department of Informatics, [4]Department of Biosciences, University of Oslo, N-0317 Oslo, Norway

Corresponding author:

Thomas Sæther, Department of Molecular Medicine, Institute of Basic Medical Sciences, University of Oslo, N-0317 Oslo, Norway. Telephone: +47-22851510. E-mail: thomas.sather@medisin.uio.no

Supplemental Table S1. Cloning primers sequences.

**Plasmid**	**Forward primer (5' – 3')**	**Reverse primer (5' – 3')**
hLXRα-DBD-mutant	CTACAATGTTCTGAGCGCCGAG GGCGCCAAGGGATTCTTCCG	CGGAAGAATCCCTTGGCGCCCT CGGCGCTCAGAACATTGTAG
ChREBPβ-exon1b-luc E-box-del	GTGCCTCCTTCTCTCCTTAGGA TGGCAGCCGCTCCTCAGGC	GCCTGAGGAGCGGCTGCCATCC TAAGGAGAGAAGGAGGCAC
ChREBPβ-exon1b-luc DR4-del	GTCTGCTCTACCCTGAGTCCTC CCTAAGCTTCTCTTCTCTTC	GAAGAGAAGAGAAGCTTAGGG AGGACTCAGGGTAGAGCAGAC
ChREBPα-LID	AATTCAGATCTATGGACTACAA GG	ATTCAAGCTTACATCACCACCT CGATGCGC

Supplemental Table S2. SYBR primers sequences.

**Gene name**	**Forward primer (5' – 3')**	**Reverse primer (5' – 3')**
Acacb	TCCTTCCAGAACTCCTCCCG	GACATGCTGGGCCTCATAGT
Chrebpα	CGACACTCACCCACCTCTTC	TTGTTCAGCCGGATCTTGTC
Chrebpβ	TCTGCAGATCGCGTGGAG	CTTGTCCCGGCATAGCAAC
Fasn	TGCACCTCACAGGCATCAAT	GTCCCACTTGATGTGAGGGG
Lpk	CAGCAGTATGGAAGGGCCAG	AGTTGCTGCTGCTGGAAGAA
Rgs16	GGGCTCACCACATCTTTGAC	TTGGTCAGTTCTCGGGTCTC
Scd1	AAAGCCGAGAAGCTGGTGAT	TACAAAAGTCTCGCCCCAGC
Tbp	GCACAGGAGCCAAGAGTGAA	TAGCTGGGAAGCCCAACTTC
Txnip	AGGGTCTCAGCAGTGCAAAC	GGCCTCATGATCACCATCTC

Supplemental Table S3. ChIP primers sequences.

**Gene name**	**Forward primer (5’ – 3’)**	**Reverse primer (5’ – 3’)**
Synthetic	ATAGGCTGTCCCCAGTGCAA	GGCTTTACCAACAGTACCGGA
Lpk	CACTCCCGTGGTTCCTGG	GGCACAGACGAGATCAGTCC
Fasn	TGCTTGGTCACACTGGAAACT	GCAGCGACACGGACCT
Scd1	GAAGCTCACCTCTTGGAGCA	GAAGTCCACGCTCGATCTCA
Txnip	ACAACAACCATTTTCCCCGC	CTCCAACCAATCAGCGAGGC

Supplemental Figure S1

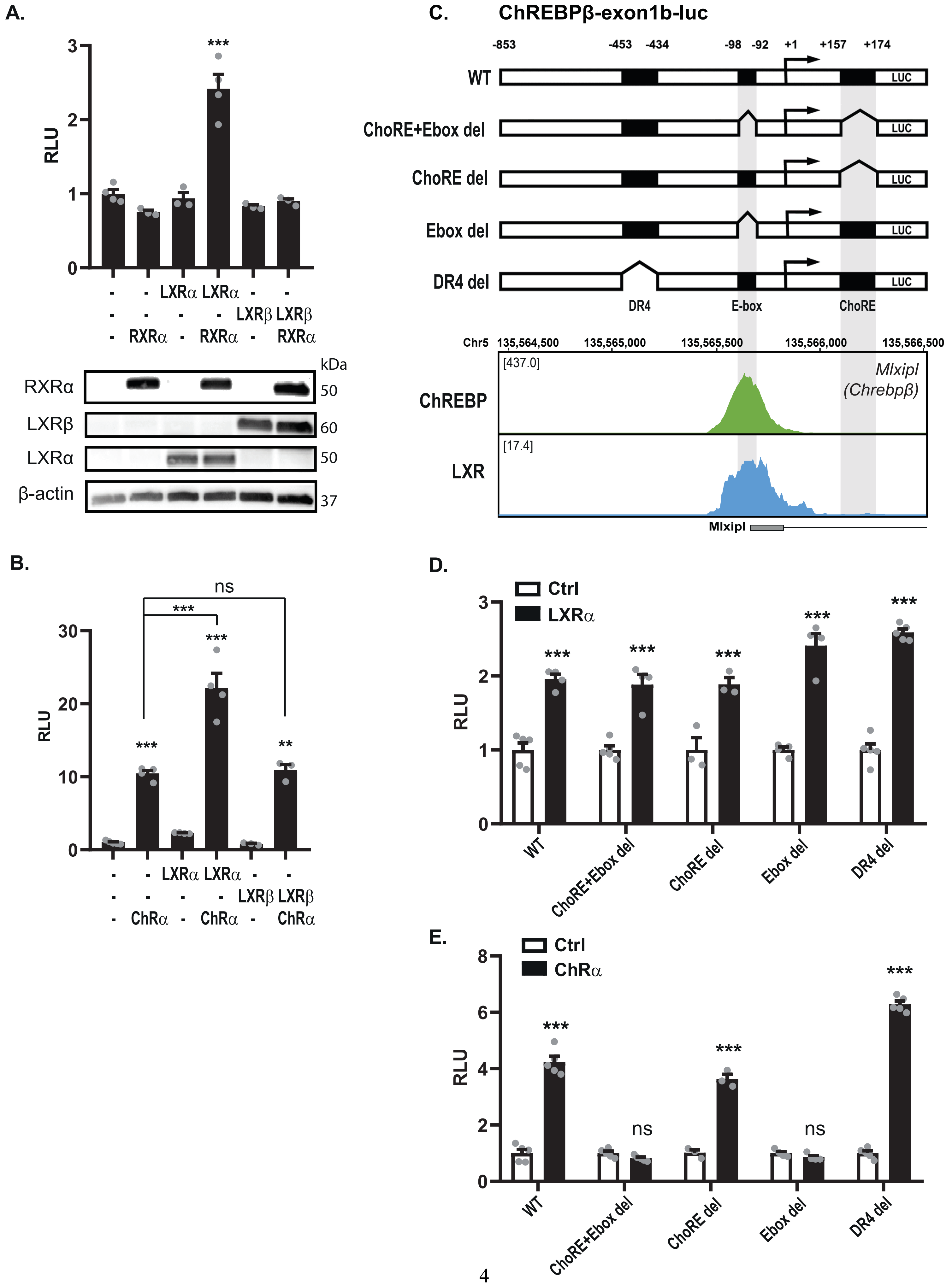

Supplemental Figure S1.

A. Top panel: Huh7 cells cultured in 25 mM glucose were transfected with a *Chrebpβ*-driven luciferase reporter, and plasmids expressing LXRα, LXRβ with or without RXRα. The Renilla lucifease reporter pRL-CMV was used as internal control. Dual luciferase reporter assays were performed 24 hours post transfection. **Bottom panel**: Corresponding lysates were immunoblotted with antibodies against LXRα, LXRβ and RXRα, and β-actin as loading control. **B.** Huh7 cells cultured in 25 mM glucose were transfected with a *Chrebpβ*-driven luciferase reporter, and plasmids expressing LXRα/RXRα or LXRβ/RXRα with or without ChREBPα/Mlxγ. The Renilla lucifease reporter pRL-CMV was used as internal control. Dual luciferase reporter assays were performed 24 hours post transfection. **C. Top panel**: Schematic representation of the *Chrebpβ*-driven luciferase reporters (Chrebpβ-exon1b-luc). **Bottom panel**: Browser view of LXR and ChREBP tracks on the *Mlxipl* (*Chrebpβ*) promoter. Square brackets indicate the scale maxima of ChIP/input ratios. DR4, direct repeat 4, potential LXRE; E-box, enhancer box; ChoRE, carbohydrate response element. **D.** Huh7 cells cultured in 25 mM glucose were transfected with the different *Chrebpβ*-driven luciferase reporters in **C**, as indicated and plasmids expressing LXRα or empty vector (Ctrl), followed by GW3965 treatment for 18 hours (10 μM).The Renilla lucifease reporter pRL-CMV was used as internal control. Dual luciferase reporter assays were performed 24 hours post transfection. **E.** Huh7 cells cultured in 25 mM glucose were transfected with the different *Chrebpβ*-driven luciferase reporters in **C**, as indicated and plasmids expressing ChREBPα or empty vector (Ctrl). The Renilla lucifease reporter pRL-CMV was used as internal control. Dual luciferase reporter assays were performed 24 hours post transfection. Data are presented as mean ± SEM (n=3-5). Significant differences are shown as $^{**}p < 0.01$, $^{***}p < 0.001$ compared to control within the same group. ns, not significant.

Supplemental Figure S2

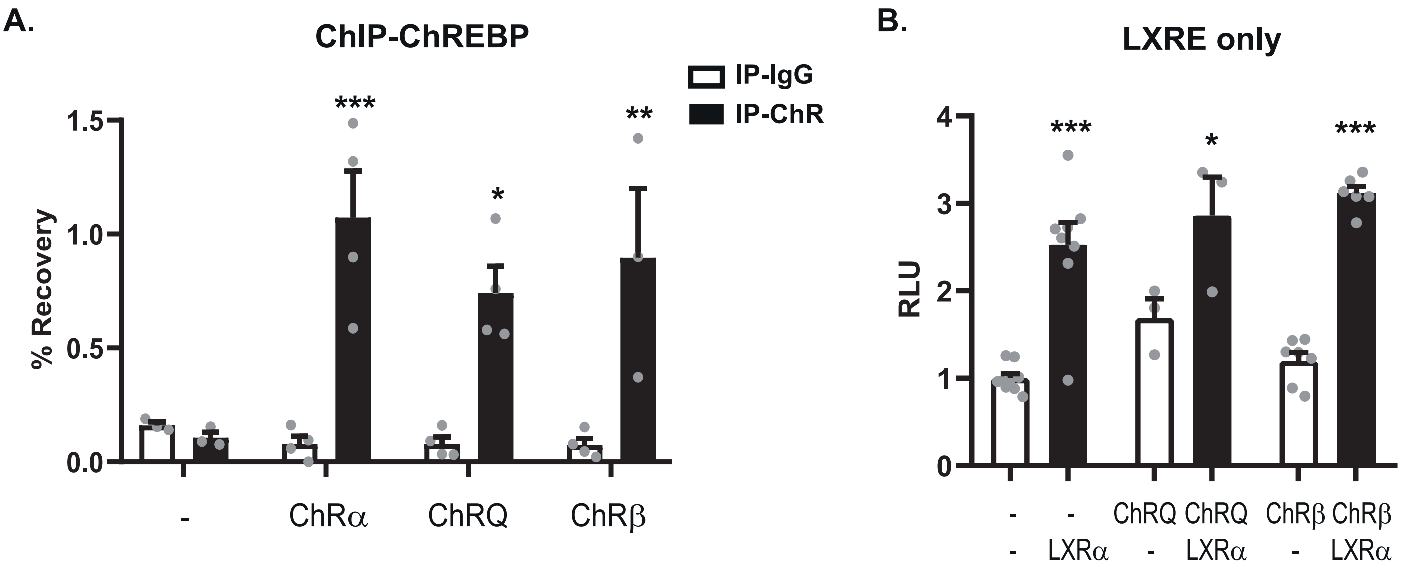

Supplemental Figure S2.

A. Huh7 cells cultured in 25 mM glucose were transfected with the with synthetic luciferase reporter containing ChoREs and LXREs and plasmids expressing ChREBPα, ChREBP-Q, or ChREBPβ, together with Mlxγ. ChREBP binding to the ChoRE were detected by ChIP using antibodies against ChREBP or IgG as control. Data are presented as mean ± SEM (n=3). Significant differences are shown as *p < 0.05, **p < 0.01 compared to IgG. **B.** Huh7 cells cultured in 25 mM glucose were transfected with the with synthetic luciferase reporter containing LXRE-only and plasmids expressing ChREBPα, ChREBP-Q, or ChREBPβ, together with Mlxγ, with or without LXRα/RXRα. The Renilla lucifease reporter pRL-CMV was used as internal control. Dual luciferase reporter assays were performed 24 hours post transfection. Data are presented as mean ± SEM (n=3-6). Significant differences are shown as ***p < 0.001 compared to control within the same ChREBP isoform transfection. ns, not significant.

Supplemental Figure S3

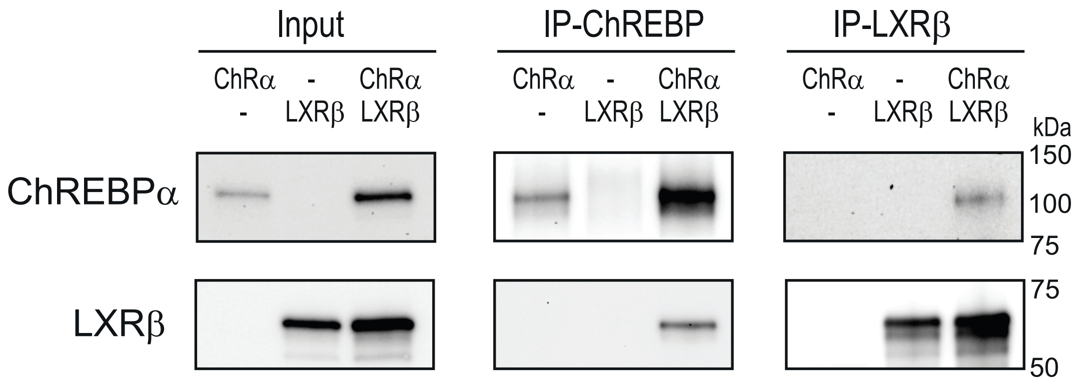

Supplemental Figure S3.

CoIP of ChREBPα and LXRβ expressed in COS-1 cells cultured in 25 mM glucose. Lysates were immunoprecipitated with ChREBP or LXRβ antibodies, and input and immunoprecipitated proteins immunoblotted with the same antibodies (n=3). One representative western blot is shown.

Supplemental Figure S4

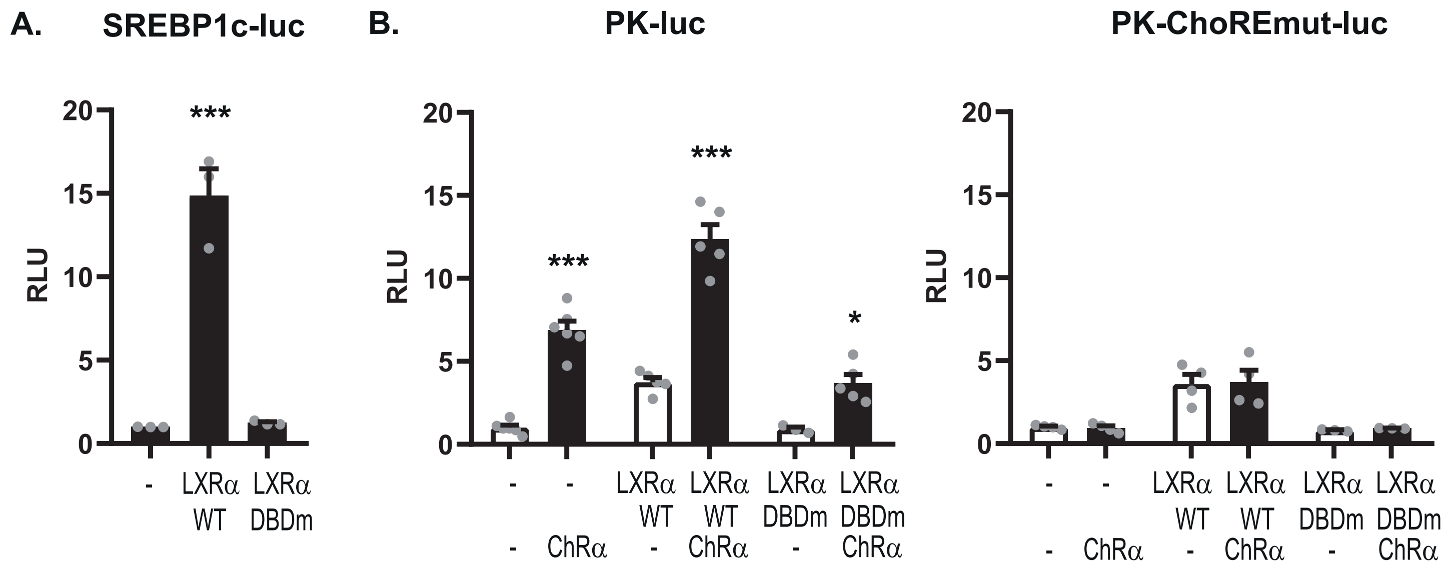

Supplemental Figure S4.

A. Huh7 cells cultured in 25 mM glucose were transfected with a *Srebp1c*-driven luciferase reporter and plasmids expressing LXRα wild-type or DNA binding mutant C115A/C118A (LXRα DBDm) together with RXRα. The Renilla lucifease reporter pRL-CMV was used as internal control. Dual luciferase reporter assays were performed 24 hours post transfection. **B.** Huh7 cells cultured in 25 mM glucose were transfected with a *Lpk*-driven luciferase wild-type reporter (PK-luc) or one where the ChoRE had been mutated (PK-ChoREmut-luc), and plasmids expressing ChREBPα/Mlxγ with or without LXRα/RXRα or LXRα DBDm/RXRα. The Renilla lucifease reporter pRL-CMV was used as internal control. Dual luciferase reporter assays were performed 24 hours post transfection. Data are presented as mean ± SEM (n=3-6). Significant differences are shown as *p < 0.05, ***p < 0.001 compared to control within the same transfection group.

Supplemental Figure S5

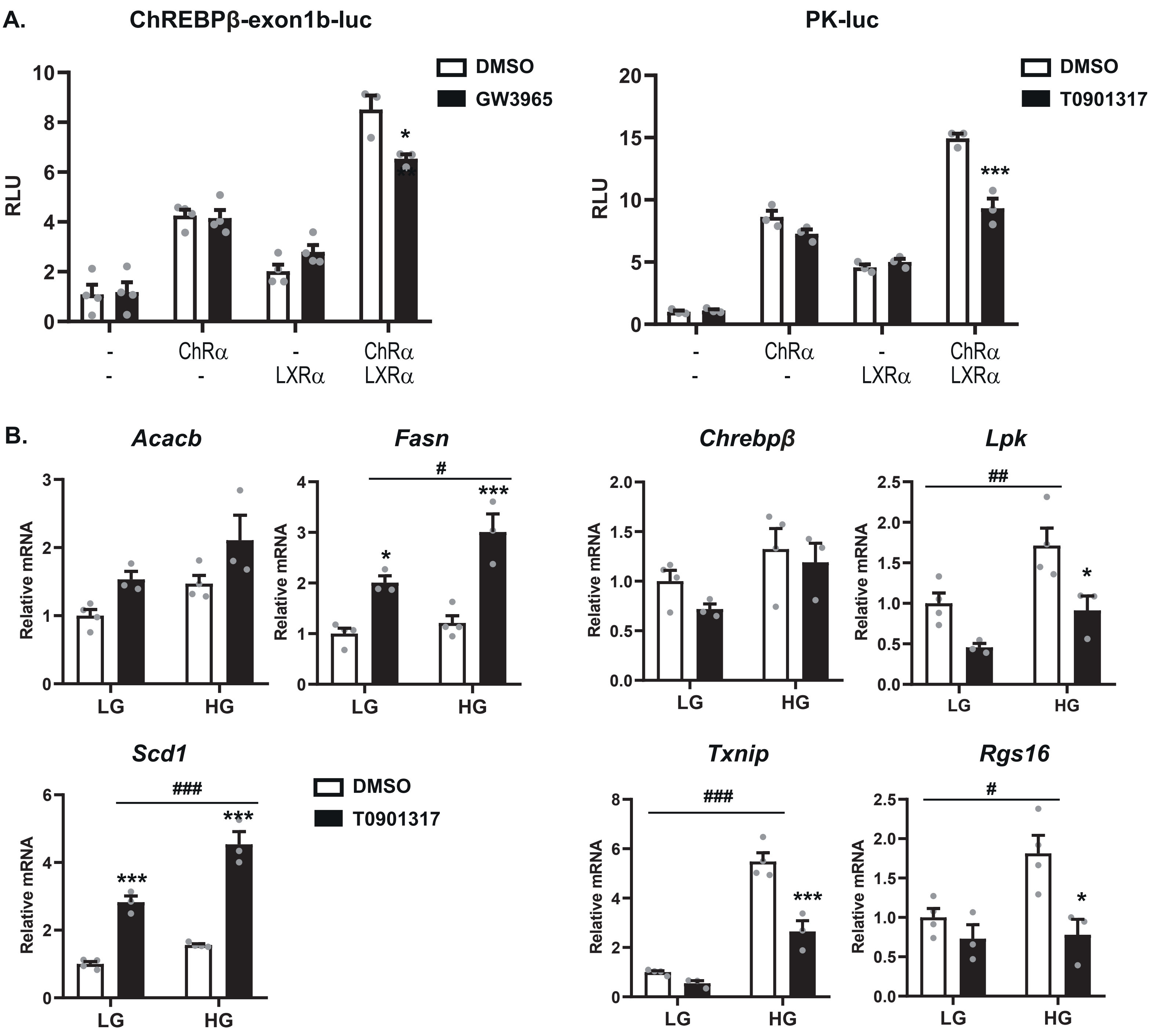

Supplemental Figure S5.

A. Huh7 cells cultured in 25 mM glucose were transfected with a *Chrebpβ* (left panel) or *Lpk*-driven luciferase reporter (right panel), and plasmids expressing LXRα/RXRα with or without ChREBPα/Mlxγ, followed by DMSO (0.1%), GW3965 (1 μM) or T0901317 (5 μM) treatment for 18 hours. The Renilla luciferase reporter pRL-CMV was used as internal control. Dual luciferase reporter assays were performed 24 hours post transfection. Data are presented as mean ± SEM (n=3-4). Significant differences are shown as $^{**}p < 0.01$, $^{***}p < 0.001$ compared to DMSO within the same group. **B.** Mouse primary hepatocytes were isolated and cultured in either 1 mM glucose (LG) or 25 mM glucose (HG) for 24 hours. For the last 18 hours the cells were treated with either DMSO (0.1%) or T0901317 (10 μM). Expression of DNL genes *Acacb*, *Fasn*, *Scd1* and ChREBP-specific target genes *Chrebpβ* (*Mlxiplβ)*, *Lpk* (*Pklr*), *Txnip* and *Rgs16* were analyzed by quantitative RT-PCR, normalized to *Tbp* and the control group set to 1. Data are presented as mean ± SEM (n=3-4). Significant differences are shown as $^{*}p < 0.05$, $^{**}p < 0.01$, $^{***}p < 0.001$ compared to DMSO within the same glucose treatment, and $^{\#}p < 0.05$, $^{\#\#}p < 0.01$, $^{\#\#\#}p < 0.001$ between LG and HG groups.

Supplemental Figure S6

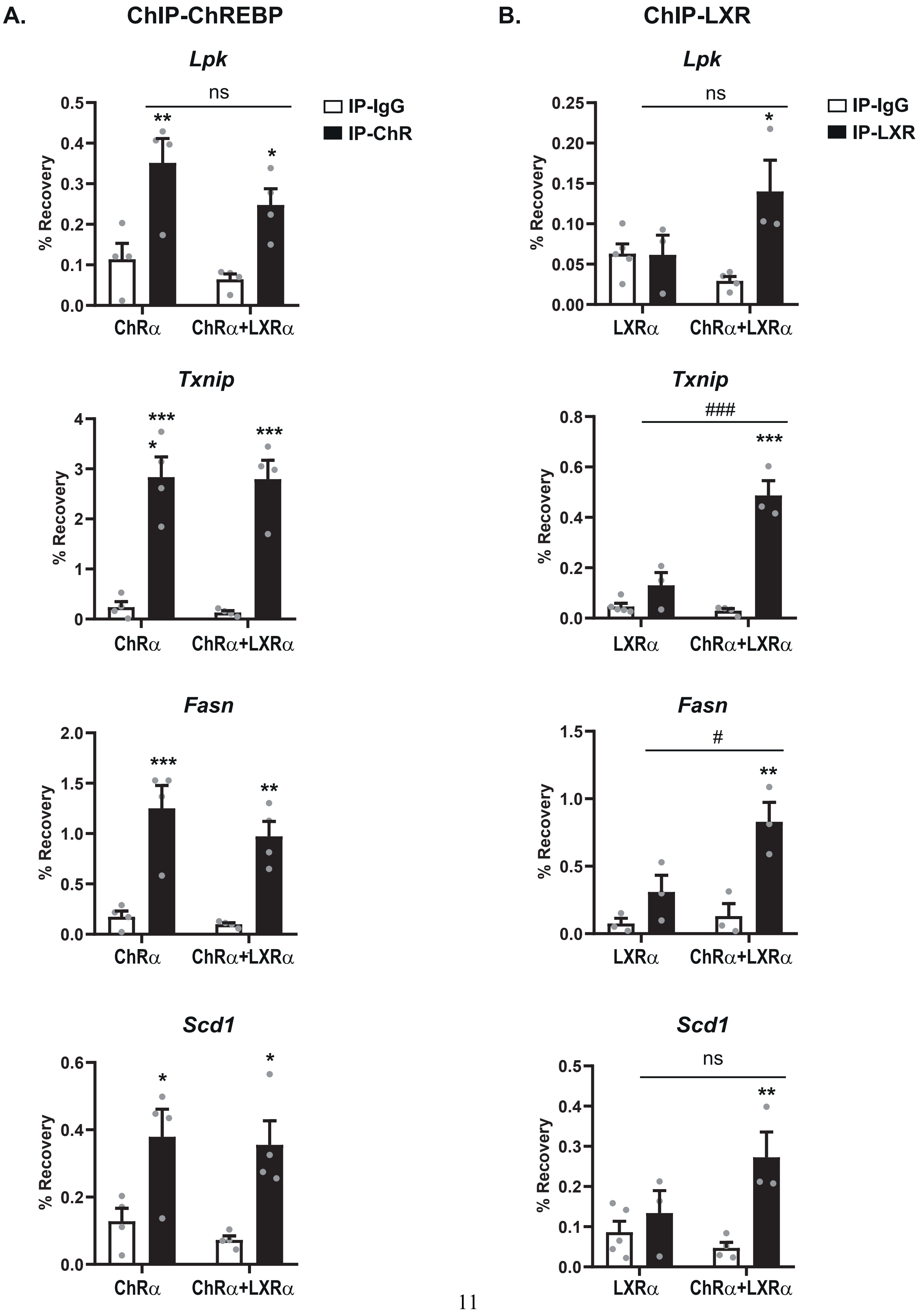

Supplemental Figure S6.

AML12 cells were transfected with ChREBPα/Mlxγ and/or LXRα/RXRα. Recruitment of (**A**) ChREBP or (**B**) LXR to the promoter region (indicated in Figure 6A left panel) of the genes *Lpk* (*Pklr*), *Txnip*, *Fasn* and *Scd1* were detected by ChIP using antibodies against ChREBP, LXR or IgG as negative control. Data are presented as mean ± SEM (n=3-5). Significant differences are shown as $*p < 0.05$, $**p < 0.01$ compared to ChIP-IgG. ns, not significant.
